# *In Vivo* Antimalarial Activity of the Solvent Fractions of Fruit Rind and Root of* Carica papaya* Linn (Caricaceae) against* Plasmodium berghei* in Mice

**DOI:** 10.1155/2017/3121050

**Published:** 2017-12-17

**Authors:** Gemechu Zeleke, Dereje Kebebe, Eshetu Mulisa, Fanta Gashe

**Affiliations:** ^1^Pharmacology Course Team, School of Pharmacy, Jimma University, P.O. Box 378, Jimma, Ethiopia; ^2^Pharmaceutics Course Team, School of Pharmacy, Jimma University, P.O. Box 378, Jimma, Ethiopia

## Abstract

**Background:**

Currently, antimalarial drug resistance poses a serious challenge. This stresses the need for newer antimalarial compounds.* Carica papaya *is used traditionally and showed* in vitro *antimalarial activity. This study attempted to evaluate* in vivo *antimalarial activity of* C. papaya *in mice.

**Methods:**

* In vivo *antimalarial activity of solvent fractions of the plant was carried out against early* P. berghei *infection in mice. Parasitemia, temperature, PCV, and body weight of mice were recorded. Windows SPSS version 16 (one-way ANOVA followed by Tukey's post hoc test) was used for data analysis.

**Results:**

The pet ether and chloroform fractions of* C. papaya *fruit rind and root produced a significant (*p* < 0.001) chemosuppressive effect. A maximum parasite suppression of 61.78% was produced by pet ether fraction of* C. papaya *fruit rind in the highest dose (400 mg/kg/day). Only 400 mg/kg/day dose of chloroform fraction of* C. papaya *root exhibited a parasite suppression effect (48.11%). But, methanol fraction of the plant parts produced less chemosuppressive effect.

**Conclusion:**

Pet ether fraction of* C. papaya *fruit rind had the highest antimalarial activity and could be a potential source of lead compound. Further study should be done to show the chemical and metabolomic profile of active ingredients.

## 1. Introduction

Even though a remarkable progress has been made, malaria remains a major health problem in Sub-Saharan Africa. It is endemic in 91 countries. The number of people infected with malaria in Sub-Saharan Africa is estimated to be 114 million in 2015. Children are especially vulnerable, accounting for more than two-thirds of global malaria deaths [1]. Malaria is also the major public health problem in Ethiopia. In Ethiopia, more than 75% of people live in malaria endemic areas, putting over 50 million people at risk of malaria. According to Federal Ministry of Health of Ethiopia 2015 report, about 1,165,843 cases of malaria were reported. In addition, it is the cause for 22,784 patients' hospital admissions [1, 2].

However, safe and effective mode of treatment is needed to control malaria and its complications. The increasing antimalarial drug resistance, insecticide resistance, and behavioral changes in Anopheles vectors threaten effective antimalarial drug therapy and malaria control and elimination [3]. Artemisinin combination therapies (ACTs) are first-line treatment for uncomplicated falciparum malaria in all endemic countries, yet partial resistance to artemisinin has emerged in the Greater Mekong Subregion [4, 5].

The famous and potent antimalarial compounds quinine (obtained from* Cinchona *species) and artemisinin (obtained from* Artemisia annua)* are derived from plants [6]. Medicinal plants have been reported for their significant antimalarial activity and remain the main focus for scientists and researchers in the development of new antimalarial agents. Phytochemical compounds including alkaloids [7], phenolic compounds [8], anthraquinones [9], and flavonoids [10] are commonly implicated for the antimalarial activity of many plants.

*Carica papaya* Linn belonging to family Caricaceae is commonly known as papaya in English, “papayyaa” in local language. Traditionally,* Carica papaya* leaves, root, and rind are used for treatment of a wide range of ailments including malaria. Many scientific investigations have been conducted to evaluate the biological activities of various parts of* Carica papaya* including their fruits, shoots, leaves, rinds, seeds, roots, or latex. The plant possessed significant biological activities such as antioxidant [11], immunomodulatory [12], anti-inflammatory [13], analgesic [14], antitumor [15], wound healing [16], and antimicrobial [17].

Previous data showed that ethanol leaf extracts of* Carica papaya* exhibited a promising inhibitory activity against the CQ-sensitive strain of* P. falciparum* [18]. In addition, the petroleum ether extract of the rind of* C. papaya *had the highest* in vitro* antimalarial activity with IC50 of 15.19 *μ*g/mL [19]. But, weak antiplasmodial activity was exhibited by the leaves and seeds of* C. papaya* [20]. There is no previous study showing* in vivo* antimalarial activity of the plant extracts.

Thus, based on* in vitro* efficacy and traditional claims, this study was aimed at evaluating* in vivo* antimalarial activity of the solvent fractions of* Carica papaya* fruit rind and root in mice.

## 2. Methods

### 2.1. Collection and Authentication of Plant Materials

The fresh* Carica papaya* fruits and roots were collected from near Jimma town, Southwest Ethiopia, in August 2016. The plant material was authenticated by a taxonomist at the Ethiopian National Herbarium, Addis Ababa University. The specimen was deposited for future reference with voucher number JU-GZ01/2016 at the National Herbarium, College of Natural Sciences, Addis Ababa University.

### 2.2. Preparation Solvent Fractions

The fresh* Carica papaya* Linn fruit rind/peel and roots were air-dried at room temperature under shade and pulverized into powder using pestle and mortar. The solvent fractions of* C. papaya* fruit rind and root were obtained by sequential soxhlet extraction with petroleum ether, chloroform, and then methanol in increasing polarity [19]. 40 grams of the powdered plant material was weighed and placed in the extraction thimble of the soxhlet apparatus. Then, about 200 ml of petroleum ether was added to the flask of the soxhlet apparatus set up. Then, the petroleum ether was heated with a temperature not exceeding 40°C to evaporate and condense into plant powder containing thimble. This extraction process was continued exhaustively until clear solution in the thimble was siphoned into the solvent flask. Then, the petroleum ether fraction was filtered with Whatman number 1 filter paper and the solvent was removed by placing in oven adjusted at a temperature less than 40°C. The marc of the petroleum ether based extraction was collected and dried at room temperature to remove petroleum ether. The dried left marc was extracted using absolute chloroform following the same procedure as described for petroleum ether extraction to get the chloroform fraction. Finally, the marc of chloroform fraction was collected and dried at room temperature. Then, the whole dried marc was further extracted with methanol with the same procedure indicated above. Each of the fractions was separately stored in screw capped vials in refrigerator until used for the study.

### 2.3. Experimental Animals

Healthy male Swiss Albino mice (8–12 weeks, weighing 25–33 grams) bred and maintained at Ethiopian Public Health Institute were used. The animals were kept in cages and housed in a standard animal house under natural 12/12 h light dark cycle at room temperature, the animal house of School of Veterinary Medicine, Jimma University. They were maintained on standard pelleted diet and water ad libitum. All mice were acclimatized for one week before the study. This study was approved by the ethical review board of college of health science of Jimma University with a reference number HRPGC/578/2015. All experiments were conducted in accordance with the internationally accepted guidelines on laboratory animal use, care, and handling [21].

### 2.4. Acute Toxicity Tests

The acute toxicity studies were conducted as per the OECD guidelines 425. Acute oral toxicity of each of the solvent fractions was evaluated in healthy female mice aged of 6–8 weeks. Five female mice were fasted for three hours and orally given a dose of 2000 mg/kg of the solvent fractions. The mice were observed for lacrimation, hair erection, behavioral change, reduction in their motor, feeding activities, and mortality for three hours and followed for 24 hours and/or 14 days [22].

### 2.5. Parasite Inoculation

Chloroquine sensitive* P. berghei *ANKA strain obtained from Ethiopian Public Health Institute and maintained at animal house facility was used. For the parasite maintenance serial passage of blood from infected mice to noninfected ones was made. A donor mouse with a parasitemia of approximately 30% was sacrificed and blood collected in a Petri-dish containing 2% trisodium citrate as anticoagulant. The blood was then diluted with 0.9% normal saline. Each mouse used in the experiment was inoculated intraperitoneally with 0.2 ml of 1 × 10^7^ * P. Berghei* infected red blood cells [23].

### 2.6. *In Vivo* Antimalarial Activity Study

*In vivo *antiplasmodial activity of the plant extract against early* P. berghei* infection was carried out according to the method described by Peter et al. (1975). Based on acute toxicity test, three doses (100, 200, and 400 mg/kg/day) were selected for the* in vivo* antimalarial study of the solvent fractions [24].

After parasite inoculation, 30 mice were randomly assigned into five groups (three treatment groups and two controls), 6 mice per group. The negative control group was treated with the vehicle 2% Tween 80. Likewise, positive control group was treated with standard drug chloroquine 25 mg/kg/day. The remaining three groups received three different doses (100, 200, and 400 mg/kg/day) of the plant extracts. The doses were administered orally at a volume of 10 ml/kg. The mice were treated after three hours of infection and continued for three days.

Weight, rectal temperature, and packed cell volume (PCV) were recorded just before infection and on day four postinfection. On day four, a thin blood film was prepared from the tail blood of each mouse. The blood films were fixed with methanol and stained with 10% Giemsa for 10 min. Blood films were examined microscopically to determine parasitemia and parasite suppression.

The mean parasitemia and % parasitemia suppression were calculated and expressed as follows [23]:

(1) %  Parasitemia=Total  number  of  Parasitized  red  blood  cellsTotal  number  of  Red  blood  cells×100,%  Parasitaemia  suppression=Parasitaemia  in  control  group−Parasitaemia  in  study  groupParasitaemia  in  control  group×100.

Moreover, each mouse was observed and monitored daily for determination of their survival time. The mean survival time (MST) for each group was calculated as follows:

(2) $$\begin{array}{c} \text{MST}=\frac{\text{Sum of survival time of mice}}{\text{Total number of mice}}. \end{array}$$

### 2.7. Determination of Packed Cell Volume

For packed cell volume (PCV) determination, blood was drawn from the tail of the different group of mice using heparinized capillary tubes before infection and on day 4 after infection.

The tubes were filled with blood up to (3/4)th of their volume and sealed at the dry end with sealing clay. The tubes were then placed in hematocrit centrifuge with the sealed end outwards and centrifuged for 5 min at 5,000 rpm [25].

(3) Packed  Cell  Volume=Volume  of  erythrocytes  in  a  given  volume  of  bloodTotal  blood  volume×100.

### 2.8. Phytochemical Screening Test

The solvent fractions of root and rind of* Carica papaya* were qualitatively screened for the presence of secondary metabolites. Thus, tests for alkaloids, flavonoids, terpenoids, phenolic compounds, tannins, saponins, anthraquinones, and cardiac glycosides were performed using standard test procedures [26, 27].

### 2.9. Data Analysis

The data was analyzed using windows software SPSS version 16 and expressed as mean ± standard error of mean (M ± SEM). Statistical significance was determined by one-way analysis of variance (ANOVA), followed by Tukey post hoc test to compare the measured parameters (parasitemia suppression, weight, rectal temperature, and survival time) within and between groups. The analysis was performed with 95% confidence interval and *p* values less than 0.05 were considered to be statistically significant.

## 3. Result

The findings from the four-day suppressive test showed that petroleum ether of* Carica papaya* fruit rind has a considerable antiplasmodial activity* in vivo *against* P. berghei* on early infections. In this study, the petroleum ether fraction of* C. papaya* fruit rind produced a dose dependent chemosuppressive effect at three doses evaluated (100, 200, and 400 mg/kg/day), with a chemosuppression of 23.03%, 34.38%, and 61.78%, respectively (Table 1). At all dose levels evaluated, the three fractions produced a statistically significant (*p* < 0.001) parasite suppression as compared to negative control. The highest chemosuppressive effect (61.78%) was exhibited by petroleum ether fraction of* C. papaya* fruit rind at 400 mg/kg/day dose.

However, a mild chemosuppression was produced by the methanol fraction of* C. papaya* fruit rind. On the other hand, the standard drug, chloroquine, caused chemosuppression of 100%. Compared to negative control, only the highest dose (400 mg/kg/day) of petroleum ether (*p* < 0.001) and chloroform (*p* < 0.05) fractions caused a statistically significant prolongation of survival time. However, the methanol fraction was not associated with significant prolongation of survival time when compared with the negative control.

As shown in Table 2,* C. papaya *root fractions produced a dose dependent and statistically significant (*p* < 0.001) chemosuppressive effect at the three doses evaluated (100, 200, and 400 mg/kg/day). The parasite suppression by petroleum ether fraction was 21.85%, 31.53%, and 43.77% for 100, 200, and 400 mg/kg/day doses, respectively. Likewise, the parasite suppression by chloroform fraction was 9.77%, 25.25%, and 48.11% for 100, 200, and 400 mg/kg/day doses. The 400 mg/kg/day dose of the ether and chloroform fractions produced the highest parasite suppression relative to the other doses.

Both 200 and 400 mg/kg/day doses of the ether fraction caused statistically significant (*p* < 0.05) survival time prolongation effect, with the mean survival time of 9.83 and 10.17 days, respectively. Similarly, 400 mg/kg/day dose of the chloroform fraction exhibited statistically significant (*p* < 0.01) survival time (10.17 ± 0.75 days) prolongation effect compared to the negative control group. This effect was still by much lower (*p* < 0.001) than that attained by chloroquine (30 ± 0.00).

**Figure 1:**
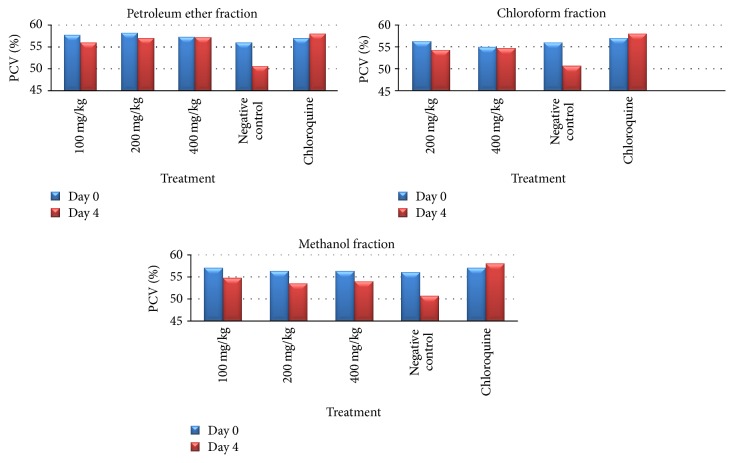
Effect of the solvent fractions of* C. papaya* fruit rind on packed cell volume (PCV) of* P. berghei* infected mice on 4-day Peter's suppression test.

Analysis of PCV change (between day 0 and day 4 after infection) shows that the higher two doses (200 and 400 mg/kg/day) of petroleum ether fraction of* C. papaya *fruit rind significantly (*p* < 0.01) prevented reduction in PCV as compared to the negative control. Likewise, only the highest administered dose (400 mg/kg/day) of chloroform fraction of* C. papaya *fruit rind significantly (*p* < 0.01) prevented reduction in PCV (Table 3, Figure 1).

The solvent fractions of* C. papaya* fruit rind exhibited a significant protection against body temperature reduction on day 4. Analysis of percent of body temperature change, between days 0 and 4, indicated that* P. berghei *infected mice treated with the three doses of the pet ether and chloroform fractions showed a statistically significant (*p* < 0.01) difference when compared to negative control (Table 4). The attenuation of the reduction in body temperature produced by 200 and 400 mg/kg/day doses of pet ether fraction of* C. papaya* fruit rind had a comparable effect to chloroquine. In chloroquine treated group, no significant change in both body temperature and PCV was observed.

On the other hand, the chloroform fraction (at all doses evaluated) of* C. papaya* fruit rind averted loss of body weight associated with infection compared to negative control. In contrast, body weight reduction caused by inoculation of the parasite was not significantly prevented by the ether fraction.

**Figure 2:**
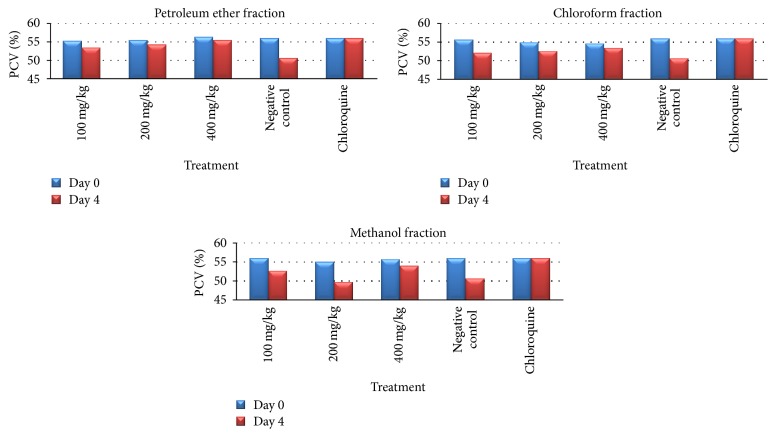
Effect of the solvent fractions of* C. papaya* root on packed cell volume (PCV) of* P. berghei* infected mice on 4-day Peter's suppression test.

Percent PCV change analysis, between days 0 and 4, revealed that the three doses (100 mg/kg with *p* < 0.05, 200 mg/kg with *p* < 0.01, and 400 mg/kg with *p* < 0.001) of petroleum ether fraction of* C. papaya* root significantly attenuated the PCV reduction compared with the negative control. Similarly, the 200 mg/kg (*p* < 0.05) and 400 mg/kg (*p* < 0.001) doses of chloroform fraction showed a statistically significant PCV protection effect unlike methanol fraction (Table 5, Figure 2).

Analysis of percent of body temperature change, between days 0 and 4, revealed that at the evaluated doses the three fractions* C. papaya* root showed a statistically significant (*p* < 0.05) difference when compared to negative control. This difference was not significant when compared with chloroquine treated group. On the other hand, the effect of petroleum ether and chloroform fractions on body weight was not dose dependent and consistent. As indicated in Table 6, the lower doses of* C. papaya* root solvent fractions produced a significant (*p* < 0.05) parasite induced body weight reduction.

### 3.1. Phytochemical Screening

In the qualitative phytochemical analysis, the solvent fractions of fruit rind and root of* Carica papaya* showed the presence of secondary metabolites such as alkaloids, flavonoids, polyphenols, tannins, and terpenoids as indicated in Table 7.

## 4. Discussion

Antimalarial drug resistance remains a major challenge and continued to emerge creating an obstacle in malaria control and elimination [5, 28]. At present, developing novel approaches and new alternative antimalarial drugs is pivotal to combat the disease [29]. From history, medicinal plants are endowed with active antimalarial compounds as artemisinin is obtained from* Artemisia annua* and quinine from* Cinchona* bark [30].* In vivo *evaluations of antimalarial activity begin with the use of the rodent malaria parasite. In addition,* in vivo *studies take into account any prodrug effect and the role of immune system in controlling malaria infection unlike* in vitro *ones [23]. Accordingly, this study evaluated the* in vivo* antimalarial activity of solvent fractions of* Carica papaya* root and fruit rind using the 4-day suppressive test, which mainly evaluates the antimalarial activity of candidates on early infections, against* P. berghei *in mice. In acute toxicity studies, the observation of no death or sign of toxicity with an oral dose of 2000 mg/kg of the fractions indicated the solvent fractions are safe for use.

In this study, all the solvent fractions* of Carica papaya* root and fruit rind exhibited a statistically significant (*p* < 0.001) and dose dependent parasite suppression effect on early infections (Tables 1 and 2). A maximum parasite suppression of 61.78% was produced by petroleum ether fraction of* Carica papaya* fruit rind in the highest dose (400 mg/kg/day), with the longest survival time compared to other fractions treated mice and negative control. Likewise, chloroform fraction of* Carica papaya *root exhibited a higher chemosuppression effect of 48.11% at 400 mg/kg/day dose. The parasite suppression exhibited by these solvent fractions was comparable with similar studies done on* D. angustifolia* [25],* Lophira alata* [31], and* Parkia biglobosa* [32]. But, a weak parasite suppression effect was exhibited by methanol fraction of both root and fruit rind of this plant.

Malaria infected mice suffer from anemia because of erythrocyte destruction, either by malaria multiplication or by spleen reticuloendothelial cell action [33]. An ideal antimalarial candidate should prevent anemia secondary to preventing hemolysis, body weight loss, and body temperature reduction in mice. In this study, a significant attenuation of PCV and body temperature reduction effect was observed by petroleum ether and chloroform fractions at 200 and 400 mg/kg doses. This was also comparable to standard drug chloroquine. The effect of the solvent fractions on body weight was variable and produced inconsistent protection. This might be due to the nature of the ingredients present in the fractions causing apatite suppression.

*In vivo* antiplasmodial activity can be classified as moderate, good, and very good if an extract displayed percent of parasite suppression equal to or greater than 50% at a dose of 500 mg, 250 mg, and 100 mg/kg body weight per day, respectively [34, 35]. Based on this classification, the petroleum ether fraction of* Carica papaya *fruit rind and chloroform fraction of* Carica papaya* root exhibited a moderate antiplasmodial activity.

In this study, pet ether fraction of* Carica papaya *fruit rind produced a promising in vivo antiplasmodial activity. This result was in agreement with* in vitro* antiplasmodial activity of pet ether (IC_50_ = 15.19 *μ*g/mL) and methanol (IC_50_ > 100 *μ*g/mL) fractions of* C. papaya *fruit rind in previous study [19]. In another study, the leaf extract of* C. papaya* produced antiplasmodial activity with IC_50_ of 46.23 *μ*g/mL [20]. This signifies that the pet ether fraction of* C. papaya *fruit rind had the highest antimalarial activity relative to the other fractions and the plant parts. This is attributed to the possible presence of the active metabolites. On phytochemical screening, terpenoids, flavonoids, alkaloids, and phenols are present in both petroleum ether and chloroform fractions that might be responsible for the observed antimalarial activity. These phytochemicals might also exert a synergistic antiplasmodial effect. But, weak antimalarial activity by the methanol fraction may be due to the presence of trace active constituents in the administered dose.

This finding may be an indicator for the presence of potential compounds with higher antimalarial activity in the petroleum ether fraction of* C. papaya *fruit rind. From this study, the active antimalarial compound found in* C. papaya *fruit rind is possibly nonpolar or semipolar in nature. Moreover, there may be a commercial potential in extracting the active compound from this plant, which grows abundantly throughout the tropics, the rind of which is discarded as waste.

## 5. Conclusion

The higher dose of pet ether and chloroform fraction of* C. papaya *fruit rind and root exhibited a moderate antiplasmodial effect with the longest survival time compared to negative control. From this finding, the petroleum ether fraction of* C. papaya *fruit rind had the highest antimalarial activity and could be targeted as potential source of lead compound in the development of new antimalarial agent. Therefore, further study should be done on pet ether fraction of* C. papaya *fruit rind to show the chemical and metabolomic profile of active ingredients from this plant.

## Figures and Tables

**Table 1:** Effect of the solvent fractions of *C. papaya* fruit rind on parasitemia and survival of *P. berghei* infected mice on 4-day Peter's suppression test.

Treatment	Dose in mg/kg/day	% parasitemia	% chemosuppression	Survival date
Petroleum ether fraction	100	40.45 ± 2.1	23.03^a3,b3,d2,e3^	8.67 ± 0.52^b3,e2^
	200	34.48 ± 4.9	34.38^a3,b3,c2,e3^	8.83 ± 0.75^b3,e2^
	400	20.08 ± 2.4	61.78^a3,b3,c3,d3^	10.33 ± 1.03^a3,c2,d2^

Chloroform fraction	100	46.92 ± 4.7	10.72^a1,b3,d2,e3^	8.33 ± 0.52^b3^
	200	39.83 ± 3.3	24.20^a3,b3,c2,e2^	8.67 ± 0.82^b3^
	400	32.77 ± 1.61	37.65^a3,b3,c3,d2^	9.5 ± 1.05^a2,b3^

Methanol fraction	100	48.25 ± 4.38	8.18^b3^	7.67 ± 0.52^b3^
	200	43.53 ± 3.25	17.16^a2,b3^	8.50 ± 0.55^b3^
	400	42.88 ± 4.88	18.39^a3,b3^	8.17 ± 0.75^b3^

Vehicle	1 ml	52.55 ± 5.6	-	7.83 ± 0.75

Chloroquine	25	0	100^a3,c3^	30.00 ± 0.00^a3^

Data are expressed as mean ± SEM; *n* = 6; ^a^compared to negative control (vehicle; 2% Tween 80), ^b^compared to chloroquine 25 mg/kg, ^c^compared to 100 mg/kg/day of the fraction, ^d^compared to 200 mg/kg/day of the fraction, and ^e^compared to 400 mg/kg/day of the fraction; ^1^*p* < 0.05; ^2^*p* < 0.01; ^3^*p* < 0.001.

**Table 2:** Effect of the solvent fractions of *C. papaya* root on parasitemia and survival of infected mice with *P. berghei *on 4-day Peter's suppression test.

Treatment	Dose mg/kg/day	% parasitemia	% chemosuppression	Survival date
Petroleum ether fraction	100	41.07 ± 2.55	21.85^a3,b3,d1,e3^	8.00 ± 0.63^b3,e2^
	200	35.98 ± 3.67	31.53^a3,b3,c1,e2^	9.17 ± 0.41^a1,b3^
	400	29.55 ± 4.42	43.77^a3,b3,c3,d2^	9.83 ± 1.33^a3,b3,c2^

Chloroform fraction	100	47.42 ± 1.94	9.77^a2,b3,d3,e3^	7.83 ± 0.41^b3,e3^
	200	39.28 ± 1.53	25.25^a3,b3,c3,e3^	8.83 ± 0.75^b3,e2^
	400	27.27 ± 3.36	48.11^a3,b3,c3,d3^	10.17 ± 0.75^a3,b3,c3,d2^

Methanol fraction	100	47.13 ± 4.39	10.31^b3,e2^	7.83 ± 0.75^b3^
	200	47.22 ± 4.97	10.15^b3,e2^	8.33 ± 1.03^b3^
	400	39.08 ± 2.76	25.63^a3,b3,c2,d2^	9.00 ± 0.89^b3^

Vehicle	1 ml	52.55 ± 5.6	-	7.83 ± 0.75

Chloroquine	25	0.00 ± 0.00	100	30.00 ± 0.00

Data are expressed as mean ± SEM; *n* = 6; ^a^compared to negative control (vehicle; 2% Tween 80), ^b^compared to chloroquine 25 mg/kg, ^c^compared to 100 mg/kg/day of the fraction, ^d^compared to 200 mg/kg/day of the fraction, and ^e^compared to 400 mg/kg/day of the fraction; ^1^*p* < 0.05; ^2^*p* < 0.01; ^3^*p* < 0.001.

**Table 3:** Effect of the solvent fractions of *Carica papaya* rind on packed cell volume (PCV) of *P. berghei* infected mice on 4-day Peter's suppression test.

Treatment group	Dose mg/kg/day	Packed cell volume		
		Day 0	Day 4	% change
Pet ether fraction	100	57.7 ± 2.34	56.0 ± 2.83	−3.02
	200	58.2 ± 2.04	57.0 ± 1.67	−2.09^a1^
	400	57.3 ± 2.42	57.2 ± 2.40	−0.29^a3^

Chloroform fraction	100	56.3 ± 1.97	53.7 ± 2.94	−5.09
	200	56.3 ± 1.50	54.3 ± 2.94	−3.85^a1^
	400	55.0 ± 1.09	54.7 ± 1.21	−0.62^a3^

Methanol fraction	100	57.0 ± 1.09	55.7 ± 2.34	−2.49
	200	56.0 ± 1.79	56.0 ± 2.19	−0.04
	400	56.3 ± 1.50	56.0 ± 2.83	−0.70

Vehicle	1 ml	56.0 ± 1.79	50.67 ± 3.26	−10.79

Chloroquine	25	57.0 ± 1.55	58.00 ± 2.10	1.69

Data are expressed as mean ± SEM; *n* = 6; ^a^compared to negative control (vehicle; 2% Tween 80); ^1^*p* < 0.05; ^3^*p* < 0.001. Day 0 = pretreatment value on day 0. Day 4 = posttreatment value on day four.

**Table 4:** Effect of the solvent fractions of *Carica papaya* rind on body temperature and weight of *P. berghei* infected mice on 4-day Peter's suppression test.

Treatment	Dose mg/kg	Temperature			Weight		
		Day 0	Day 4	% change	Day 0	Day 4	% change
Pet ether fraction	100	37.1 ± 0.07	36.6 ± 0.33	−1.37^a3^	27.3 ± 1.03	26.3 ± 1.97	−4.15
	200	37.1 ± 0.13	36.9 ± 0.43	−0.64^a3^	22.0 ± 1.90	23.5 ± 1.22	6.41^a3^
	400	37.1 ± 0.07	36.9 ± 0.25	−0.41^a3^	26.0 ± 0.89	25.8 ± 1.17	−0.72^a2^

Chloroform fraction	100	37.2 ± 0.14	35.7 ± 0.29	−1.27^a3^	24.2 ± 2.14	23.5 ± 2.17	−3.01
	200	37.1 ± 0.12	37.0 ± 0.32	−0.36^a3^	26.5 ± 2.51	26.7 ± 3.20	0.30^a2^
	400	37.1 ± 0.08	36.9 ± 0.13	−0.41^a3^	24.7 ± 1.97	25.0 ± 1.55	1.23^a2^

Methanol fraction	100	37.1 ± 0.13	35.5 ± 0.34	−1.65^a3^	25.5 ± 1.05	24.2 ± 1.33	−5.66^a1^
	200	37.1 ± 0.15	35.7 ± 0.29	−1.09^a3^	23.8 ± 0.98	24.3 ± 1.21	1.91^a3^
	400	37.0 ± 0.08	35.6 ± 0.55	−1.29^a3^	24.5 ± 1.22	24.7 ± 1.21	0.62^a3^

Vehicle	1 ml	37.1 ± 0.14	34.05 ± 0.78	−5.89	25.33 ± 2.16	22.3 ± 2.25	−13.66

Chloroquine	25	37.1 ± 0.39	37.30 ± 0.32	0.41^a3^	33.3 ± 5.05	34.3 ± 5.35	3.16

Data are expressed as mean ± SEM; *n* = 6; ^a^compared to negative control (vehicle; 2% Tween 80); ^1^*p* < 0.05; ^2^*p* < 0.01; ^3^*p* < 0.001. Day 0 = pretreatment value on day 0. Day 4 = posttreatment value on day four.

**Table 5:** Effect of the solvent fractions of *Carica papaya* root on PCV, body temperature, and weight of *P. berghei* infected mice on 4-day Peter's suppression test.

Treatment group	Dose mg/kg/day	Packed cell volume		
		Day 0	Day 4	% change
Petroleum ether fraction	100	55.3 ± 2.94	53.5 ± 1.22	−3.45
	200	55.5 ± 1.52	54.3 ± 3.44	−2.34^a1^
	400	56.3 ± 1.97	55.5 ± 1.52	−1.51^a3^

Chloroform fraction	100	55.7 ± 1.50	52.17 ± 2.23	−6.81
	200	55.0 ± 1.09	52.7 ± 1.63	−4.47
	400	54.7 ± 1.03	53.5 ± 2.17	−2.28^a2^

Methanol fraction	100	56.0 ± 1.26	51.7 ± 1.50	−8.41
	200	55.0 ± 1.09	49.8 ± 2.23	−10.50
	400	55.7 ± 1.50	54 ± 1.79	−3.11

Vehicle	1 ml	56.0 ± 1.79	50.7 ± 3.27	−10.79

Chloroquine	25	56.0 ± 1.55	56.0 ± 2.10	1.69

Data are expressed as mean ± SEM; *n* = 6; ^a^compared to negative control (vehicle; 2% Tween 80); ^1^*p* < 0.05; ^2^*p* < 0.01; ^3^*p* < 0.001. Day 0 = pretreatment value on day 0. Day 4 = posttreatment value on day four.

**Table 6:** Effect of the solvent fractions of *C. papaya* root on body temperature and body weight of *P. berghei* infected mice on 4-day Peter's suppression test.

Treatment group	Dose mg/kg/day	Temperature			Weight		
		Day 0	Day 4	% change	Day 0	Day 4	% change
Petroleum ether fraction	100	37.2 ± 0.15	35.9 ± 0.23	−0.68^a3^	24.7 ± 3.26	24.2 ± 3.12	−2.20^a1^
	200	37.1 ± 0.12	37.0 ± 0.13	−0.18^a3^	28.3 ± 2.25	27.5 ± 1.76	−3.04^a1^
	400	37.1 ± 0.09	37.0 ± 0.19	−0.22^a3^	22.2 ± 1.94	20.8 ± 2.99	−7.36

Chloroform fraction	100	37.1 ± 0.10	35.5 ± 0.38	−1.70^a3^	20.2 ± 1.17	19.5 ± 1.05	−3.52^a1^
	200	37.2 ± 0.07	36.9 ± 0.10	−0.63^a3^	20.5 ± 1.38	19.8 ± 1.72	−3.75^a1^
	400	37.1 ± 0.12	36.9 ± 0.16	−0.50^a3^	22.7 ± 1.37	21.5 ± 1.52	−5.62

Methanol fraction	100	37.1 ± 0.12	34.5 ± 0.35	−1.60^a3^	21.2 ± 1.60	22.5 ± 1.64	1.39
	200	37.1 ± 0.18	34.6 ± 0.27	−1.37^a3^	21.0 ± 0.89	18.8 ± 1.94	−12.32
	400	37.1 ± 0.12	35.7 ± 0.29	−1.09^a3^	20.3 ± 1.63	19.8 ± 1.72	−2.60^a1^

Vehicle	1 ml	37.1 ± 0.13	34.0 ± 0.78	−5.89	25.3 ± 2.16	22.3 ± 2.25	−13.66

Chloroquine	25	37.1 ± 0.39	37.3 ± 0.32	0.41^a3^	33.3 ± 5.05	34.3 ± 5.35	3.16^a1^

Data are expressed as mean ± SEM; *n* = 6; ^a^compared to negative control (vehicle; 2% Tween 80); ^1^*p* < 0.05; ^3^*p* < 0.001. Day 0 = pretreatment value on day 0. Day 4 = posttreatment value on day four.

**Table 7:** Qualitative phytochemical screening of the solvent fractions of fruit rind and root of *Carica papaya*.

Secondary metabolites	*C. papaya* fruit rind fractions			*Carica papaya* root fractions		
	Pet ether	Chloroform	Methanol	Pet ether	Chloroform	Methanol
Alkaloids	+	+	+	+	+	+
Flavonoids	+	+	−	+	+	−
Polyphenols	+	+	+	+	+	+
Tannins	+	+	+	+	+	+
Terpenoids	+	+	+	+	+	
Cardiac glycosides	−	−	−	−	−	−
Saponin	−	−	+	−	−	−

*Note*. + indicates the presence and − indicates absence of particular metabolites.

## Additional Points

*Availability of Data and Materials*. The complete data are all contained within the paper and are also available from the corresponding author on reasonable request.
